# High-content live-cell multiplex screen for chemogenomic compound annotation based on nuclear morphology

**DOI:** 10.1016/j.xpro.2022.101791

**Published:** 2022-10-26

**Authors:** Amelie Tjaden, Robert T. Giessmann, Stefan Knapp, Martin Schröder, Susanne Müller

**Affiliations:** 1 Institute of Pharmaceutical Chemistry, Goethe University Frankfurt, Max-von-Laue-Str.9, 60438 Frankfurt, Germany; 2 Structural Genomics Consortium, BMLS, Goethe University Frankfurt, Max-von-Laue-Str. 15, 60438 Frankfurt, Germany; 3 Bayer AG, Research & Development, Pharmaceuticals, 13353 Berlin, Germany; 4 Institute for Globally Distributed Open Research and Education (IGDORE), Berlin, Germany

**Keywords:** Cell biology, Cell-based assays, Cancer, High throughput screening, Microscopy

## Abstract

Well-characterized small molecules enable the study of cell processes and facilitate target validation. Here, we describe a high-content multiplex screen to investigate cell viability over 48 h, which can be combined with investigating phenotypic features, such as tubulin binding and mitochondrial content, as initial cellular quality control of diverse compounds. The protocol is on a live-cell basis and easily adaptable and scalable. It details cell preparation, compound handling, plate layout configuration, image acquisition with the CQ1, and data analysis using the CellPathfinder software.

For complete details on the use and execution of this protocol, please refer to Tjaden et al. (2022).

## Highlights

• A fast and flexible multiplex assay for compound annotation
• Evaluate cell properties in live-cell mode
• Machine learning techniques to optimize high-content data evaluation
• Easily adaptable and scalable for different phenotypic features

## Abstract (editor highlights)

Publisher’s note: Undertaking any experimental protocol requires adherence to local institutional guidelines for laboratory safety and ethics.

## Graphical abstract

**Figure:**
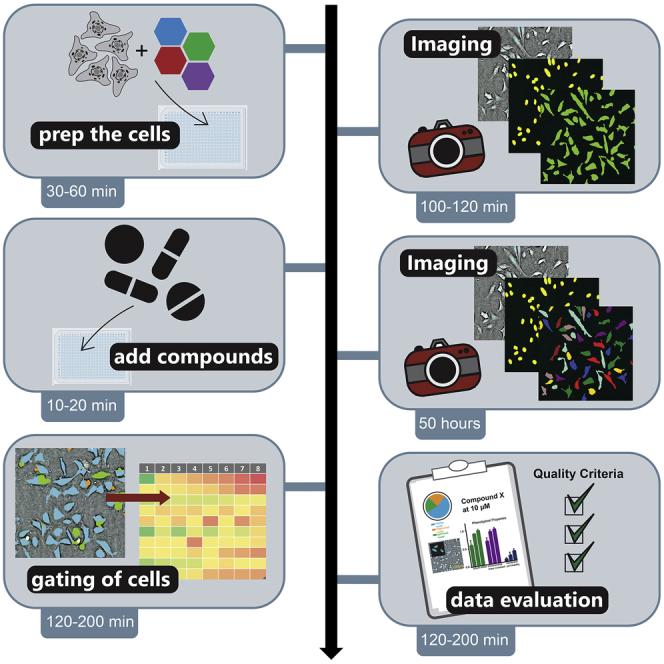

## Before you begin

Here, we describe a high content multiplex screen, able to characterize generic effects regarding basic cell properties and cell viability in a multi-dimensional way, using phenotypic characterization to annotate compounds on their suitability for further phenotypic and mechanistic screening.

The present protocol describes the workflow for 135 test compounds, screened at two concentrations (1 and 10 μM) in addition to 6 control compounds in a single 384 well plate, measured for a total of 48 h at 4 different time points.

The protocol below describes the specific steps for testing osteosarcoma cells (U-2 OS). However, we have adapted this protocol to other cell lines including human embryonic kidney cells (HEK293T) and human fibroblasts (MRC-9) (see problem 1).

### Culture cell lines

**Timing: 2 weeks**

1. Prior to the experiment, U-2 OS cells were cultured over two weeks in modified Dulbecco’s Modified Eagle’s medium (DMEM) plus L-Glutamine (4 mM) and high glucose (4.5 g/L D-Glucose), supplemented with 10% fetal bovine serum (FBS) and 1% Penicillin (100 U/mL)/Streptomycin (100 μg/mL) in a corning T-75 cell culture flask.
  a. The cells should be incubated at 37°C and 5% CO_2_ partial pressure in a suitable incubator.
  b. Constant cell growth was obtained by passaging the cells two to three times a week.
    i. The cells should be passaged when they reach 70%–80% confluence.
    ii. Cells were detached from the culture flask using trypsin (0.025% Trypsin and 0.01% EDTA), followed by quenching with the modified DMEM and diluted at a ratio of 1:30 (2–6 × 10^5^ cells) in a new culture flask with fresh, modified DMEM.
  c. In our experience, U-2 OS cells of passage 5–35 yield reproducible results.

### Optimize cell seeding density

**Timing: 48 h**

**Figure 1:**
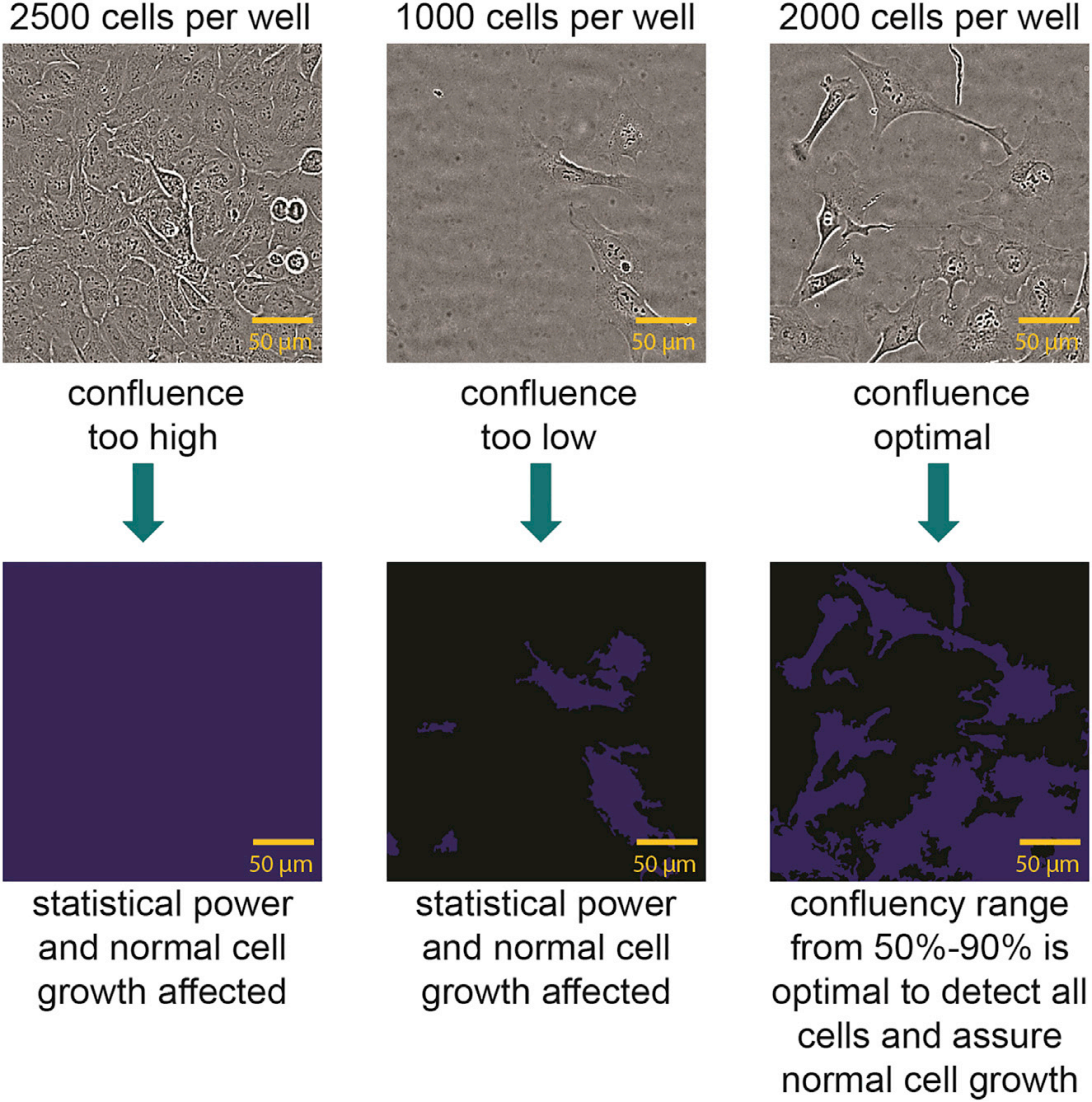
Confluence display of U-2 OS cells Brightfield image of U-2 OS cells stained after 48 h using the Cellcyte X live-cell imaging system. Images show U-2 OS after different cell seeding concentrations (2500 cells/well, 1000 cells/well and 2000 cells/well) and the detection of the confluence using the implemented Cellcyte X analysis software.

The described protocol is optimized and validated for immortalized and primary adherent cell lines. Due to the autofocus mechanism of the imager, suspension cells, as well as 3D cultures, will require additional optimization steps and may not be suitable for this protocol (see problem 1). Live cell imaging is best accomplished when cells in the wells have a confluence ranging from 50 to 90%. High confluence can result in difficulties in the segmentation of individual cells. Low confluence can affect the statistical power of the data (see problem 2) and the overall cell behavior. Depending on the length of the experiment, cell densities should be adapted accordingly. The starting confluence should be chosen such that robust segmentation of individual cells can still take place after 48 h i.e., cells should not be overgrown at that time point. We conducted an experiment before the start of the experiment to determine the best seeding concentration of U-2 OS cells for optimal cell segmentation and cell growth (see Figure 1). We therefore recommend that the confluence at the first time point should be more than 40% and after 48 h the confluence should not exceed 90%.

2. Optimize cell seeding density by setting up a preliminary experiment with varying cell densities and monitor their confluence over 48 h.
  a. Wash the cells cultured in a T75 culture flask once with 5 mL PBS buffer.
  b. Detach the cells using 3 mL trypsin and determine the cell count and viability of the cells using trypan blue 0.4% solution in a Neubauer chamber or an automated cell counter such as the TC20 Automated Cell Counter (Bio-Rad).
    i. Mix 10 μL cells and 10 μL trypan blue and add to a counting slide for automated cell counting.
    ii. Count the unstained (live) and stained (dead) cells.
    iii. Viability of the cells should be more than 95%.
  c. Dilute the cells into 6 different concentrations ranging from 2,500 cells/well to 1,000 cells/well in steps of 250 cells/well.
  d. Pipet 50 μL/ well of diluted cells according to the layout shown in Table 1 in a clear bottom, black, 384-well plate.

**Table 1:** Layout to determine optimal cell seeding density

384 plate	1	2	3	4	5	6	7	8
A	Buffer	buffer	buffer	buffer	buffer	buffer	buffer	buffer
B	Buffer	2500	2250	2000	1750	1500	1250	1000
C	Buffer	2500	2250	2000	1750	1500	1250	1000
D	Buffer	2500	2250	2000	1750	1500	1250	1000
E	Buffer	2500	2250	2000	1750	1500	1250	1000
F	Buffer	2500	2250	2000	1750	1500	1250	1000
G	Buffer	2500	2250	2000	1750	1500	1250	1000
  e. Create 3 mL of a stock solution with 50,000 cells/mL for a final count of 2,500 cells/well.
    i. For 2,500 cells pipet 50 μL of stock solution in row 2 B to G.
    ii. For 2,250 cells pipet 45 μL of stock solution and 5 μL of media in row 3 B to G.
    iii. For 2,000 cells pipet 40 μL of stock solution and 10 μL of media in row 4 B to G.
    iv. For 1,750 cells pipet 35 μL of stock solution and 15 μL of media in row 5 B to G.
    v. For 1,500 cells pipet 30 μL of stock solution and 20 μL of media in row 6 B to G.
    vi. For 1,250 cells pipet 25 μL of stock solution and 25 μL of media in row 7 B to G.
    vii. For 1,000 cells pipet 20 μL of stock solution and 30 μL of media in row 8 B to G.
    viii. Pipet 100 μL/well of PBS or any other buffer in the outer wells to minimize evaporation variations (see problem 3).

3. Image the plate one day after seeding (time point 0 h) and 48 h after the first imaging. For this step any light microscope can be used. Here we used the Cellcyte X Live-cell imaging system.
  ***Note:*** The first imaging will represent the baseline time point of the later experiments. The second and third imaging step is important to validate the confluence after 48 h.
  a. Image the plate once 24 h after seeding and 48 h after time point 0 h using the brightfield channel at 10× magnification.

4. Determine confluence levels at both time points (Figure 1).
  a. Either use the software implemented in the microscope used or any other software that can detect the confluence level of the cells using the brightfield channel. We used the implemented Cellcyte X analysis software.
    i. Train the software with 5–10 example wells.
    ii. Use wells from different columns to increase the variability of the confluence levels.
    iii. Analyze the whole plate and check by eye on 5–10 other wells, to see if the mask detected all cells.
  b. The confluence at the first time point should be sufficient for normal cell growth.
  c. Confluence after second time point should not exceed 90%.
5. Select the best concentration of cells, at which you can see normal cell growth (Musa et al., 2013) and the confluence after 48 h does not exceed 90%.

**CRITICAL:** Ensure that the cells are seeded at an appropriate density and are evenly distributed within the wells to avoid malfunction of the autofocus and ensure statistical evaluability (see problem 6). To make sure the cells are evenly distributed when attaching to the plate, leave the plate at room temperature for 30 min before placing it in the incubator. For best measurement results, the plate should be left in the incubator for a few minutes before being imaged.

### Preparation of compounds and plate layout

**Timing: 5 min per compound**

The compounds of interest as well as specific reference compounds necessary for the experiment can be prepared in advance. If using the Echo 550 automated liquid handler, we recommend preparing a source plate and a “pick list”. The reference compounds are necessary to train the machine learning algorithm. For each cell property, at least one reference compound must be tested. As part of the assay development, we tested the described reference compounds in U-2 OS cells (Tjaden et al., 2022) and confirmed their suitability to generate a training dataset (see data analysis CellPathfinder software).

6. All compounds should be dissolved and/or diluted in a suitable solvent such as DMSO to a 1000-fold higher concentration than the final assay concentration. This ensures a minimal final concentration of 0.1% DMSO which has been found to produce no measurable effect (Brayton 1986) on the proliferation of U-2 OS cells.

***Note:*** If other cell lines are used, the DMSO sensitivity should be tested beforehand (see problem 4).

7. If using an automated acoustic liquid handler, such as an Echo 550 (LabCyte) the compounds should be added to a compatible source plate, e.g., 384-well LDV Source plate (Labcyte).
  a. Add a minimum 3 μL of 1000× compound solution into each well of the source plate.
  b. Centrifuge the LDV source plate once at 500 × *g* for 3 min.
  c. To minimize compound contamination, the plates should be sealed with DMSO resistance seals (VWR) after each usage.
  d. The plates can be stored at -20°C over a longer time period (for at least 2 years on average).
    i. One compound plate can be used multiple times, when stored correctly.
    ii. Before reusing the plate, ensure that the compounds are completely thawed. We recommend leaving the compound plate at least one hour at room temperature before pipetting. Also, precipitation of compounds should be checked by eye, because precipitation of compounds within the source plate can lead to false negative results (see problem 4).
    iii. Check also if there is enough compound solution left for your experiment. You can check that using the survey detection function included in the Echo550 Labcyte software.
    iv. To minimize absorption of the compound into the wall of the storage plate, use polypropylene plates or other plates recommended for compound storage.
8. For the analysis, use the following reference compounds at a final concentration of 10 μM: staurosporine, paclitaxel, milciclib, daunorubicin, digitonin and berzosertib (see Table 2).
  a. Dissolve reference compounds in DMSO to a final concentration of 10 mM.
  b. Add a minimum 3 μL of each of these reference compounds to an individual well of your LDV source plate when pipetting with the Echo 550.

**Table 2:** Reference compounds with mode of actions and gating steps they are used for

Reference compound	Mode of action	Used for what gating step
staurosporine	promiscuous kinase inhibitor (Bruno et al., 1992)	apoptotic cells, pyknosed nuclei
paclitaxel	targets tubulin/no disassembly of mitotic spindle (Wang et al. 2000)	Change of tubulin structure
milciclib	CDK inhibitor (Sanchez-Martinez et al., 2015)	Increase of mitochondrial mass
daunorubicin	anthracycline antibiotic, intercalation of DNA strands, ROS production (Al-Aamri et al., 2019)	Increase of mitochondrial mass, fragmented nuclei, apoptotic cells
digitonin	Detergent (Styrt et al. 1985)	Permeabilization of plasma membranes
berzosertib	ATR/ATM-inhibitor (Fokas et al., 2012)	Hoechst high intensity objects
9. Prepare a pick list by using the “Pick list” program for the Echo 550 according to the manufacture’s manual (manual can be found here: https://www.agilent.com/cs/library/usermanuals/Public/G5415-90026_DDUG_EN.pdf).
  a. A possible layout of the pick list is shown in Table 3.

**Table 3:** Layout Pick list for Echo550 Liquid Handling System

Source plate name	Source plate well	Destination plate name	Destination plate well	Transfer volume (nL)	Compound description
Source Plate 1	A01	Destination Plate 1	B02	50	Compound 1
Source Plate 1	A02	Destination Plate 1	B03	50	Compound 2
Source Plate 1	A03	Destination Plate 1	B04	50	Compound 3
Source Plate 1	A04	Destination Plate 1	B05	50	Compound 4
10. For an example of a pick list refer to Table S1 (Zenodo: https://zenodo.org/record/7092795#.YyhponZByUk).
11. Add the compounds as well as the reference compounds to your pick list so that they will be added to your destination plate.
  a. The reference compounds should be added in technical quadruplicates (see Figure 2) to get a sufficient validation set for the analysis protocol (see data analysis CellPathfinder software).

**Figure 2:**
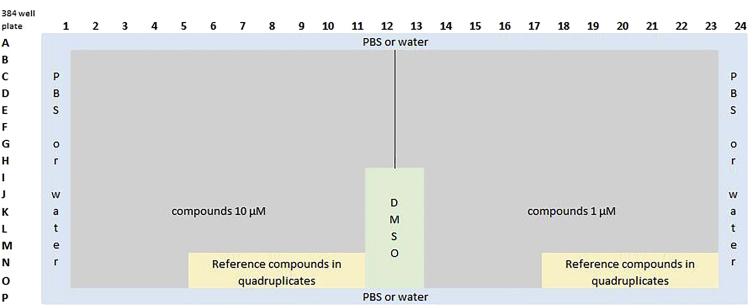
Possible Layout of one 384-well plate to test 135 compounds at two different concentrations
  b. Compounds should be pipetted to the destination plate at the concentration of interest in a final volume of 50 μL. The standard protocol assesses compounds at 10 μM and 1 μM. Thus, 50 nL and 5 nL of inhibitor stock solution should be transferred, respectively (see example in Table S1 (Zenodo: https://zenodo.org/record/7092795#.YyhponZByUk)). Due to the large dilution factor, the volume of 45 nL DMSO can be neglected and a final concentration of 0.1 vol% of DMSO is not affecting cell (Brayton 1986) proliferation.
  c. Compounds are tested in technical duplicates.
  d. 5%–10% of the plate should be “blank” wells filled with DMSO 0.1% i.e., by transferring 50 nL of DMSO per well. This is similar to the dilution factor for compounds from 10 mM -> 10 μM.
12. A possible layout of the plate can be found in Figure 2.
13. Save your picklist as “.csv” on your local storage space.

**CRITICAL:** Compounds with limited solubility in DMSO can precipitate in the source wells and will therefore not be dispensed to the cells. Pay attention to minimize freeze-thaw-cycles, because this can affect the stability of the compound solutions. Test DMSO solubility of the compound stock solutions before testing the compound. If not soluble in DMSO, try diluting in water and pipet by hand or with ECHO LabCyte protocol for aqueous solvents (384LDV_aq). (See problem 4).

### Determination of channels and scan parameters for imaging protocol

**Timing: 1 h**

For image acquisition, the protocol for the CQ1 can be found under the name “Measurement protocol for the Multiplex assay using the CQ1 and files containing the settings to operate the CQ1 microscope with Momentum” at Zenodo: https://doi.org/10.5281/zenodo.6394521. However, for every change in the assay protocol (different plate, different cell line, different conditions etc.) or when using a different microscope, the protocol should be adjusted accordingly.

14. Here are the standard settings in detail:
  a. Magnification: A 10× objective is used, as it will capture a large surface area while retaining resolution to capture cell morphology. Higher magnifications like 20× can also be used to obtain a more detailed image, but with the consideration that fewer cells can be imaged. Lower magnification of 2× might not detect detailed phenotypic properties.
  b. Five channels are used for image acquisition: Ex 405 nm/Em 447/60 nm, 500 ms, 50%; Ex 561 nm/Em 617/73 nm, 100 ms, 40%; Ex 488/Em 525/50 nm, 50 ms, 40%; Ex 640 nm/Em 685/40, 50 ms, 20%; brightfield, 300 ms, 100% transmission.
  c. Focus and Z-stacks: The area where the Z-stacks will be set can vary from plate to plate. Use 7 Z-stacks for optimal cell imaging with a total of 55 μm spacing to ensure the compensation of potential plate variations and generation of a robust readout. If using the autofocus for your experiment, it may happen that the microscope will sometimes focus on dust and will lose focus (see problem 6), so we recommend to set the focus area for 384-well plates with a height of 14,4±0,1 mm at -15.5 μm for the lowest (-20.2 μm) and highest focus of 39.7 μm (51.6 μm), respectively.

***Note:*** All parameters should be adjusted when details in the set-up are changed e.g., when other cells, plates or other dyes are used. Also, the time frame can freely be adapted depending on the experiment. For viability studies an incubation time of at least 24 h is recommended.

**CRITICAL:** Use the same plates that are implemented in the CQ1 measuring software. If the plate differs, the focus area might not be as accurate as intended (see problem 6). The specific plate parameters must be implemented in the CQ1 software (refer to the manufacturer’s manual, which can be found here: https://www.ibios.uni-osnabrueck.de/index.php?cat=Light%20Microscopy/Yokogawa%20CQ1&file=CQ1_User_Manual.pdf).

### Preparation of the data analysis workflow

**Timing: 2 h**

15. Install Python 3 on your computer, preferentially via the distribution Anaconda.
  a. Anaconda can be downloaded from https://www.anaconda.com.
  b. Follow the Getting started instructions at https://docs.anaconda.com/anaconda/user-guide/getting-started/ to open the Anaconda Prompt.
  c. Note the path displayed in the Prompt. This will be named “root path” in this protocol.
16. To set up your Python environment and prepare the analysis scripts, visit Zenodo: https://doi.org/10.5281/zenodo.6325622 and follow the instructions given.

***Note:*** The exact version and specific DOI of the software you will be using by checking the “Versions” box in Zenodo, and cite the Zenodo record correspondingly in your work (e.g., v1.0.1 of the scripts has the DOI: Zenodo: https://doi.org/10.5281/zenodo.6327204).

## Key resources table

**Table:** 

REAGENT or RESOURCE	SOURCE	IDENTIFIER
**Chemicals, peptides, and recombinant proteins**		

staurosporin	Selleckchem	Cat#: S1421
paclitaxel	Selleckchem	Cat#: S1150
digitonin	Invitrogen	Cat#: BN2006
milciclib	Selleckchem	Cat#: S2751
daunorubicin	Selleckchem	Cat#: S3035
berzosertib	Selleckchem	Cat#: S7102
Hoechst33342	Thermo Scientific	Cat#: 62249 https://www.thermofisher.com/order/catalog/product/62249
BioTracker™ 488 Green Microtubule Cytoskeleton Dye	EMD Millipore	Cat#: SCT142 https://www.merckmillipore.com/DE/de/product/BioTracker-488-Green-Microtubule-Cytoskeleton-Dye,MM_NF-SCT142
MitoTracker red	Invitrogen	Cat#: M22425 https://www.thermofisher.com/order/catalog/product/M22425
Annexin V Alexa Fluor 680 conjugate	Invitrogen	Cat#: A35109 https://www.thermofisher.com/order/catalog/product/A35109
Trypan blue 0.4%	Thermo Fisher Scientific	Cat#: 15250061 https://www.thermofisher.com/order/catalog/product/15250061

**Critical commercial assays**		

alamarBlue ^TM^ Cell Viability Reagent	Invitrogen	Cat#: DAL1025

**Deposited data**		

raw images and processed images	BioImage Archive	S-BIAD145
protocol establishment information	Tjaden et al.	https://doi.org/10.20944

**Experimental models: Cell lines**		

U-2 OS cells	ATCC	HTB-96™

**Software and algorithms**		

CQ1 microscope software	Yokogawa	v 1.04.03.01
CellPathfinder Software v3.04.02.02	Yokogawa	N/A
Python v3.9	Python Software Foundation	N/A
Microsoft Excel v16.0.4266.1001	Microsoft	N/A
GraphPad Prism v8.4.3	GraphPad Software	N/A
Cellcyte Studio	CYTENA	v2.6.0
Scripts for data analysis	Python	Zenodo: https://doi.org/10.5281/zenodo.6325622
CellPathfinder analysis protocols	Yokogawa	Zenodo: https://doi.org/10.5281/zenodo.6415330

**Other**		

CQ1 microscope	Yokogawa	N/A
384-well cell culture microplate, PS, f-bottom, μClear®	Greiner	Cat#: 781091
ECHO® 550 Acoustic Liquid Handler	Labcyte	N/A
ECHO® source plate	Labcyte	Cat#: P.05525
Cytomat2C24 incubator	Thermo Scientific	N/A
TC20 Automated Cell Counter	Bio-Rad	https://www.bio-rad.com/de-de/product/tc20-automated-cell-counter?ID=M7FBG34VY
CELLCYTE X^TM^	CYTENA	v6.1.1
DMSO resistance seals	VWR	391-0642
Supplemental Material		Zenodo: https://zenodo.org/record/7092795#.YyhponZByUk

## Materials and equipment

This protocol should be adaptable to other confocal microscopes, which have similar imaging capacities than the Yokogawa CQ1. However, the image acquisition and analysis would need to be adapted accordingly.

For data analysis, other software, such as the open-source software “deepImageJ” with an implemented deep learning function (https://deepimagej.github.io/deepimagej/) or other built-in software can be used, though the described analysis will no longer be applicable and further data reformatting steps might be necessary.

***Optional:*** As an alternative to a liquid handling system, manual pipetting was tested. The volume and concentration of the chemicals and compounds have to be adapted accordingly to transfer volumes accurately.

The CQ1 can also be implemented in an automation system like Momentum v5.3.

This protocol has been validated against three different adherent cell lines, immortalized and non-immortalized (Tjaden et al., 2022).

## Step-by-step method details

### Addition of fluorescent dyes and cell seeding

**Timing: 45–60 min**

These steps describe the preparation of cells, the seeding and the following addition of the fluorescent dyes to the cells.

1. Dilution of U-2 OS cells for seeding of one 384 well plate.
  a. Wash the cells in a T75 flask once with 5 mL PBS buffer.
  b. Detach the cells using 3 mL trypsin and determine the concentration and viability of the cells using trypan blue 0.4% solution.
    i. Mix 10 μL cells and 10 μL trypan blue and add to a counting slide for automated cell counting.
    ii. Put the counting slide into the TC20 Automated Cell Counter.
    iii. Count the unstained (live) and stained (dead) cells.
    iv. Wait till the results appear on the Cell Counter screen.
    v. Viability of the cells should be higher than 95%.
  c. Dilute the cells to 3 × 10^4^ cells/mL and a final volume of 16 mL/384 well plate or the cell concentration determined in your preliminary experiment for a confluence ranging from 50%–90% after 48 h.

***Note:*** For a 384-well plate format and 50 μL per well, the cell concentration range is typically between 2–4 × 10^4^ cells/mL (1,000–2,000 cells/well). Depending on the cell type and experimental setup this can be adapted using the steps described above see optimize cell seeding density.

2. Addition of the fluorescent dyes to the cells.
  a. Add to the 16 mL cell suspension prepared in step 1c:
    i. 57 μL of a 16.23 μM stock of Hoechst33342 (final assay concentration of 60 nM).
    ii. 8 μL of a stock of BioTracker™ 488 Green Microtubule Cytoskeleton Dye.
    iii. 12 μL of a 100μM stock of Mitotracker red (final assay concentration of 75 nM).
    iv. 93 μL of a stock of Annexin V Alexa Fluor 680 conjugate.
      ***Note:*** Using different fluorescent dyes than the ones mentioned here, can potentially lead to bleed-through problems. For the here described protocol, the overlapping of fluorescence emission spectra is neglectable for all dyes but the MitoTracker Red and Annexin V Alexa Fluor 680. However, this overlap does not influence the analysis, since the excitation maxima of these two dyes are well separated and the gating. See also (Tjaden et al., 2022).
  b. Mix gently by inverting the cell suspension.
  c. Transfer 50 μL of the cell suspension with fluorescent dyes prepared in step 2 to the corresponding wells of the 384 well plate using a multichannel pipet and a reagent reservoir. The plate layout can be found in Figure 2.
  d. Pipett 100 μL PBS buffer or water in the outer wells as indicated in Figure 2.
    ***Note:*** This step helps avoiding edge effects due to evaporation (see problem 3).

3. Remove any air bubbles that may have been formed during pipetting (see Figure 3). This step can be performed using an ethanol bottle where a straw-like nozzle is positioned above the ethanol surface within the bottle. Gently press air on the surface of the cell dilution in the wells to remove the air bubbles.

**Figure 3:**
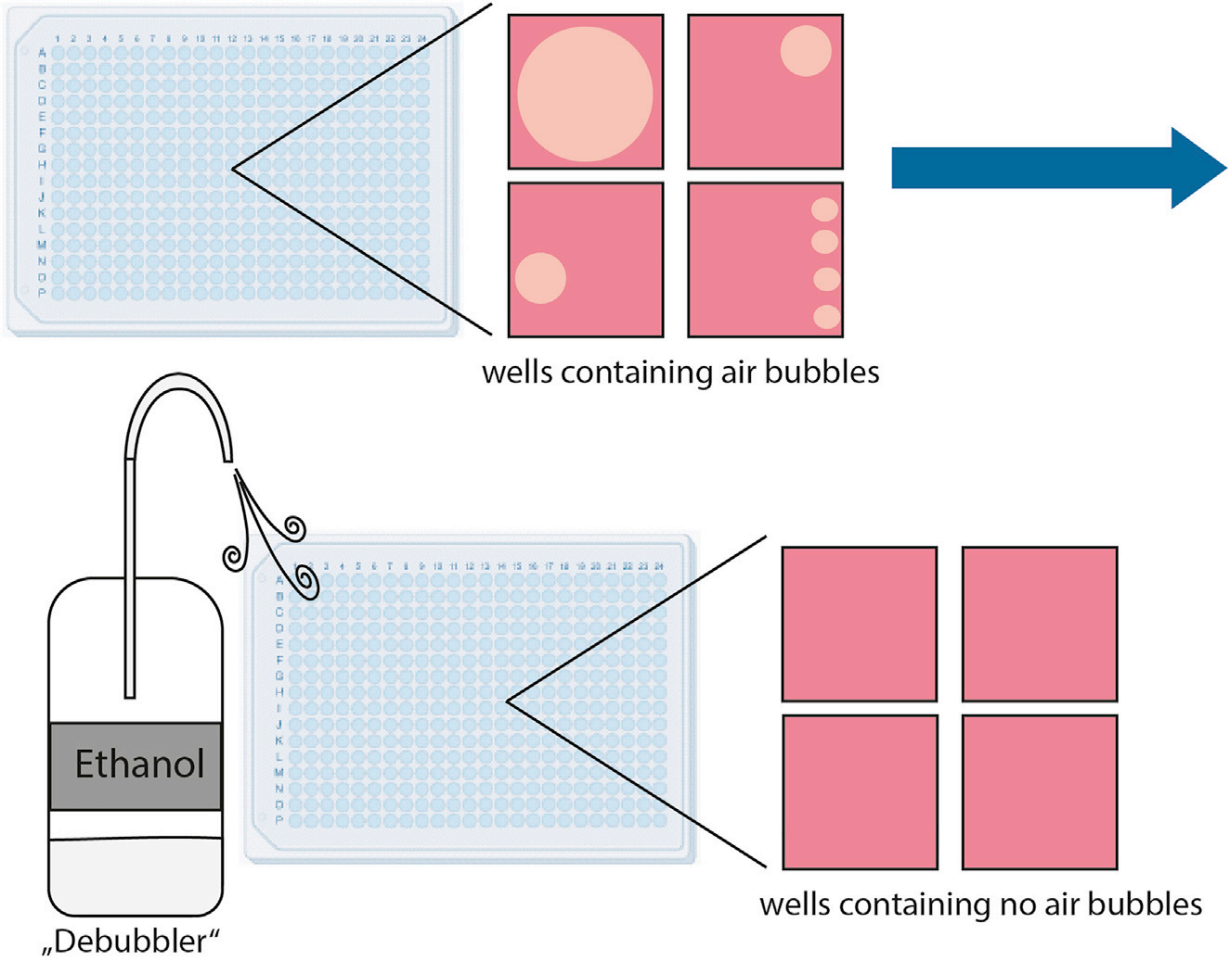
Debubbler Example illustration of air bubble removal.
4. Put a lid on the plate prepared in steps 3–6 and leave it for 30 min under the cell culture hood at room temperature to allow reattachment of the U-2 OS cells to the plate.
5. Incubate the plate for 18–24 h at 37°C and 5% CO_2_.

Figure 3**Debubbler**. Example illustration of air bubble removal.

**CRITICAL:** Ensure an even and reproducible distribution of cells within the wells. Image acquisition should ideally be performed in the center of the well to ensure the best coverage of the well area. Uneven distribution of cells within a well can at a later point impede the statistical analysis (see problem 5).

**Pause point:** The following steps will be conducted the next day.

### Image acquisition of non-treated cells

**Timing: 3–4 h**

This step describes the image acquisition of the non-treated cells. The resulting data serve as a baseline for the statistical normalizations (see evaluate your data).

6. Switch on the CQ1 microscope and the separate laser module.
  a. Wait 3 min for the laser to heat up.
7. Ensure that the environmental controls regulating temperature, CO_2_ partial pressure as well as the water reservoirs in the CQ1 microscope and the plate incubator are functioning.
  a. The temperature of the CQ1 microscope should be kept stable at 37°C.
  b. The partial pressure of CO_2_ should be set to 5%.
  c. The humidity should be set to 100% to avoid evaporation (see problem 3).
8. Place the plate prepared in steps 3–6 in the CQ1 and open the imaging software using either the associated automation unit or manual handling.
9. Open the measuring protocol, described in the section determination of channels and scan parameters for imaging protocol.
10. Assess if the measuring protocol provides focused images of sufficient intensity in all channels.
  a. Choose randomly approximately 10 wells spread over the entire plate.
  b. Test if the focus area and channel parameters can detect the cells and fluorescent signals of the different channels (see problem 6).
11. Measure the plate once with non-treated cells using the described protocol.
12. Put the plate into an incubator at 37°C and 5% CO_2_ until compound treatment.

***Note:*** The time between the measurement and the compound treatment should be as short as possible.

### Compound treatment

**Timing: 30–60 min**

This step describes compound treatment of the cells.

13. Use the Echo® source plate (see preparation of compounds and plate layout) prepared in step 7 to add the compounds to your plate.
14. Use the ‘‘Pick list’’ program in the Echo® 550 liquid handler system to aliquot the correct volumes of the inhibitors and DMSO into each reaction wells in the assay plate (see 9–13 in preparation of compounds and plate layout).
  a. Open the “Pick list” program for the Echo 550 system on your computer.
  b. Select “new” to create a new protocol.
  c. Enter for the source- and destination plate the right Sample Plate Format (384LDV) and the right Sample Plate Type.
    i. For DMSO as a solvent use 384LDV_DMSO as plate type.
  d. Now you can import your picklist, which should be saved as “.csv” on your local storage space.
    i. Check your layout again on “plate preview”.
15. After adding the compounds, centrifuge the plate for 3 min at 100 × *g*.

### Image acquisition treated cells

**Timing: 24–48 h**

This step describes the image acquisition of the treated cells over a defined time.

16. Make sure that the CQ1 microscope is prepared as described in steps 8 and 9 of Image Acquisition of non-treated cells and place the plate in the CQ1.
17. Open the measuring protocol used for the acquisition of the non-treated cells (see image acquisition of non-treated cells).

***Note:*** For image acquisition of treated cells, the same measuring protocol should be used as is for non-treated cells. It is important that the same cell area will be imaged and therefore robust baseline values can be calculated.

18. Test the measuring protocol with the inserted cell plate.
  a. Therefore, scan 3–5 wells to test if the focus area of the initial baseline measurement is still appropriate (see problem 6). If the CQ1 cannot detect the cells, the plate might not be placed properly into the machine or there was a problem while pipetting (e.g., air bubbles occurred).
  b. Define Scan Schedule and Frequency of your testing. Here, we measure the plate 12 h, 24 h and 48 h after compound treatment (see Figure 4).

**Figure 4:**
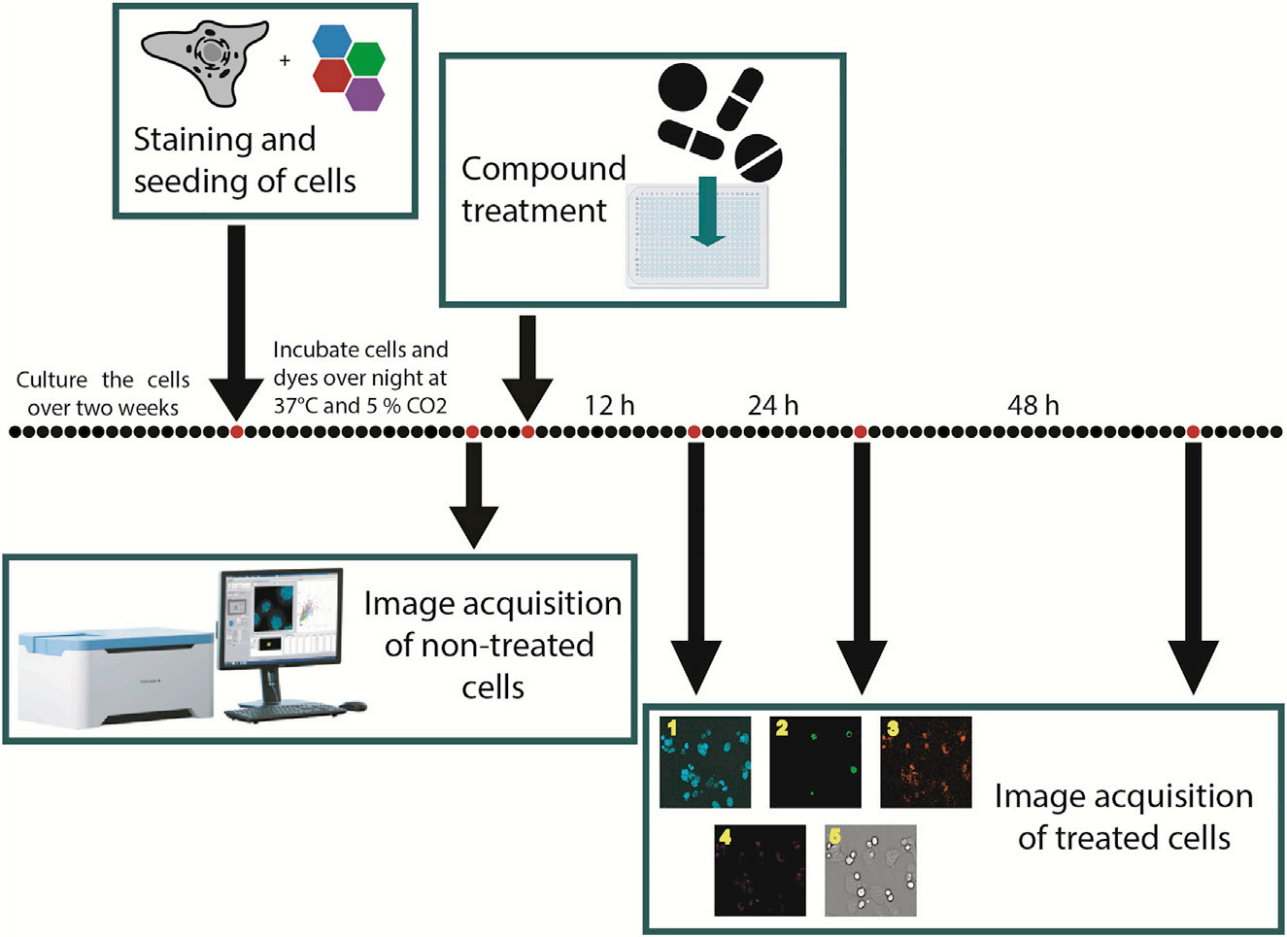
General time schedule Schemed time schedule of one plate, which is measured before compound treatment and 12 h, 24 h and 48 h after compound treatment using the CQ1 confocal microscope.
19. To free instrument time of the CQ1 microscope, the plate can be also transferred to an incubator (37°C, 5% CO_2_).

### Data analysis CellPathfinder software

**Timing: 4–5 h**

This step describes the image analysis using the CellPathfinder software (Yokogawa) and is specific to the CQ1 microscope. Similar high-content microscopes with their own built-in software for analysis or open-source analysis software should be compatible with this protocol, however will require hard- and software related adaptation. For more comprehensive instructions regarding the Pathfinder software, consult the Yokogawa’s product specialists and technical documents. The resulting data and processed images will be evaluated afterwards (see evaluate your data) to obtain a phenotypical annotation of each tested compound. The protocol we used to analyze U-2 OS cells “CellPathfinder_AnalysisProtocol_U2OS_Multiplex” can be found here: Zenodo: https://doi.org/10.5281/zenodo.6415330.

***Note:*** The generated protocol is specific to the cell type and magnification. Experiments with different components will require generating a new protocol.

20. Open the CellPathfinder software (see Figure 5A).

**Figure 5:**
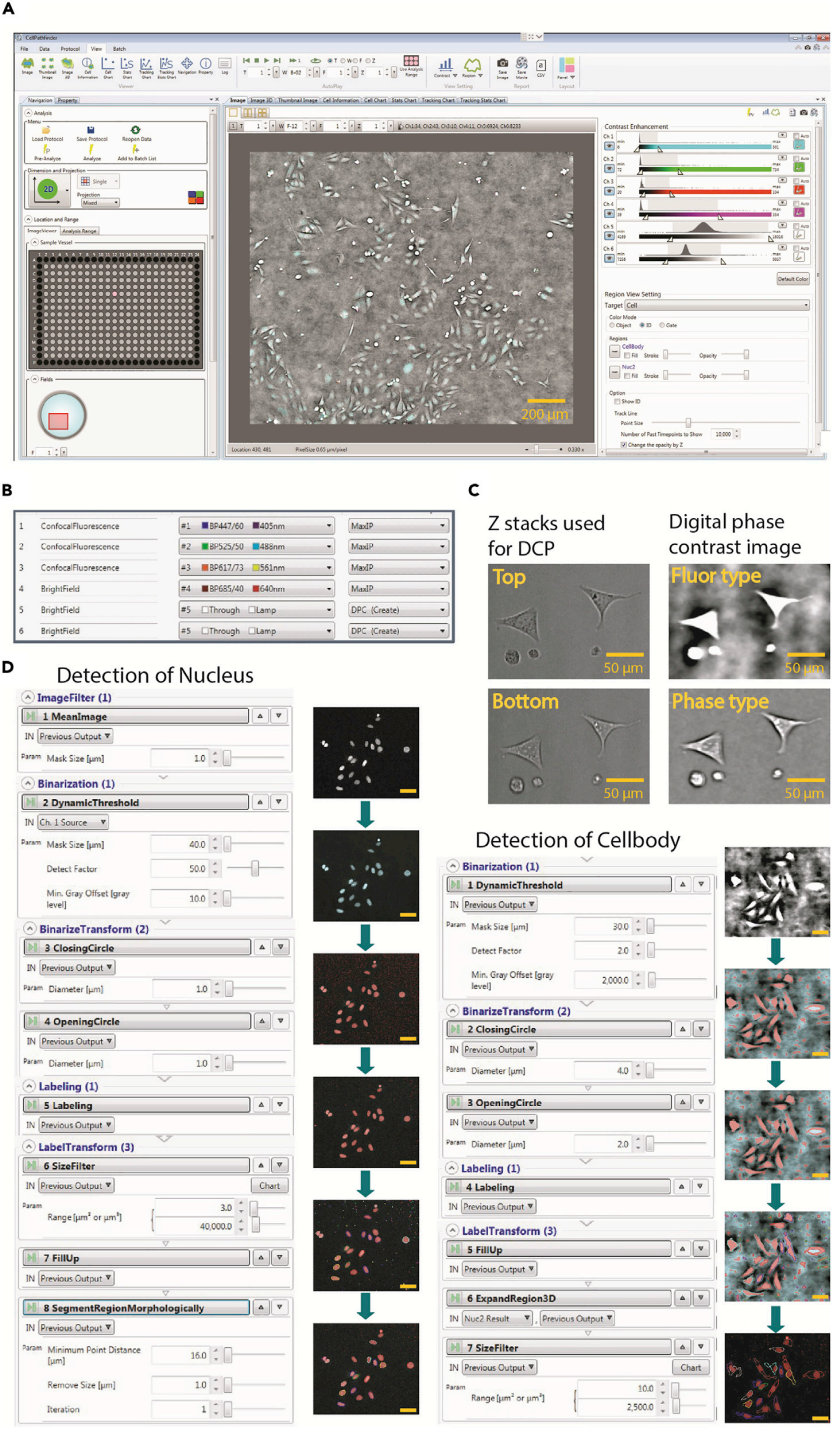
Protocol generation in CellPathfinder software (A) Operation window for CellPathfinder software. (B) Channels used for analysis. (C) Z-stacks used for digital phase contrast (DCP) and DCP as fluo- and phase type. (D) Algorithm used to detect nucleus and cell body, as well as example images.
21. In the Data tab you can see the measurement details of your experiment.
22. Open the data you want to analyze first.
  a. Open the folder on your computer where you saved the CQ1 measurement data.
  b. Select the data that you want to analyze first.
  c. Convert the CQ1 image data by clicking “convert”.
23. It is recommended to create an analysis for each cell line individually.
24. Open the Protocol tab and click on “Load” and select the analysis protocol for U-2 OS cells “CellPathfinder_AnalysisProtocol_U2OS_Multiplex”.Now you can go step by step through the parameters by clicking on the process buttons at the top of the operation window (see Figure 5A).
25. Open the “Channels” section. We are using 6 different channels (CH) (see Figure 5B), based on our study design. The brightfield is used twice.
  a. CH1: Hoechst33342 (DNA detection): Ex 405 nm/Em 447/60 nm.
  b. CH2: BioTracker™ 488 Green Microtubule Cytoskeleton Dye (tubulin stain): Ex 488/Em 525/50 nm.
  c. CH3: MitoTracker red (mitochondrial mass detection): Ex 561 nm/Em 617/73 nm.
  d. CH4: Annexin V (apoptosis marker): Ex 640 nm/Em 685/40.
  e. CH5: brightfield, DCP Fluor Type Simple Mode, Contrast 0.06.
  f. CH6: brightfield, DCP Phase Type Simple Mode, Contrast 0.04.
26. Channels 5 and 6 are set on DCP to create the best digital phase contrast (see Figure 5C).
  ***Note:*** The “fluortype” setting of the digital phase contrast (DPC) in CH5 provides better segmentation of the cells against the background while the phase setting in CH6 can be used to generate high contrast cell images for visual inspection or publication figures.
  a. For both digital phase contrast channels, two Z stacks should be used to be overlaid and get the best contrast image to detect cellular shape. Here, we used Top Z 5 and Bottom Z 3. Depending on the plate, these Z stacks might differ.

27. For Object assembly open “Object”. Here we created 2 different objects (“Nucleus” and “Cell body”).
  a. “Nucleus”: Ex 405 nm/Em 447/60 nm, Finder: Nuclear, Recognition: Advance.
  b. “Cell body”: brightfield, Finder: Cell, Recognition: Advance.
28. A process based on the defined objects was created (Figure 5D). The aim of the algorithm is to detect cellular shape “Cell body”, based on the brightfield and cell nuclei “Nucleus” based on fluorescent signal of the Hoechst channel. For the Cell body we used the brightfield channel with the fluor-type (CH5).
29. The Nuclei and the Cells are linked in the “Link” section.
  a. Included nucleus in cell body.
  b. One in one (1:1).
30. To proceed with the protocol in the CellPathfinder software, you have to first pre-analyze your data. This step includes image processing and object detection and is inevitable to later use the machine learning-based functions for the cellular gating. For an easier and faster workflow, you can pre-analyze just wells with reference compounds important for training of the machine learning (ML)-based functions as well as non-treated cells (see preparation of compounds and plate layout).
  a. The pre-analyzing of important wells should secure the detection of cell bodies as well as cell nuclei. Check if the cell bodies and nuclei are correctly detected in 5–10 example wells.
  b. Click on “Pre-Analyze” and select the wells including the reference compounds (N5 to N12 and O5 to O12) (see preparation of compounds and plate layout).
31. After pre-analysis, the ML based gating algorithm can be trained by opening the “Gate” section.
32. All features we used can be found in the Table S2 (Zenodo: https://zenodo.org/record/7092795#.YyhponZByUk).
33. The machine learning based workflow of the analysis is shown in Figure 6.
  ***Note:*** To detect the phenotypic changes based on healthy nuclei, a tree principle is used. For a detailed explanation on the gating principle and all features used, see (Tjaden et al., 2022).
  a. The cells will be gated first into the categories Hoechst High Intensity Objects “HighIntObj” or normal cells “Normal”.
  b. All cells that are gated “Normal” will then be gated into cells containing a healthy nucleus “HealthyNuc”, a pyknosed nucleus “PyknoNuc” or a fragmented nucleus “FragNuc”.
  c. A pyknosed nucleus can either appear due to mitosis or apoptosis. Thus, cells with pyknosed nuclei will be gated further into “mitosis” or “apoptosis”.
  d. To investigate the phenotypic properties of interest (tubulin effect, mitochondrial mass increased, membrane permeabilized), cells showing a healthy nucleus will be gated further in “tubulin effect” or “tubulin normal”, “mito mass high” or “mito mass normal” and “membrane permeabilized” or “membrane intact”.

**Figure 6:**
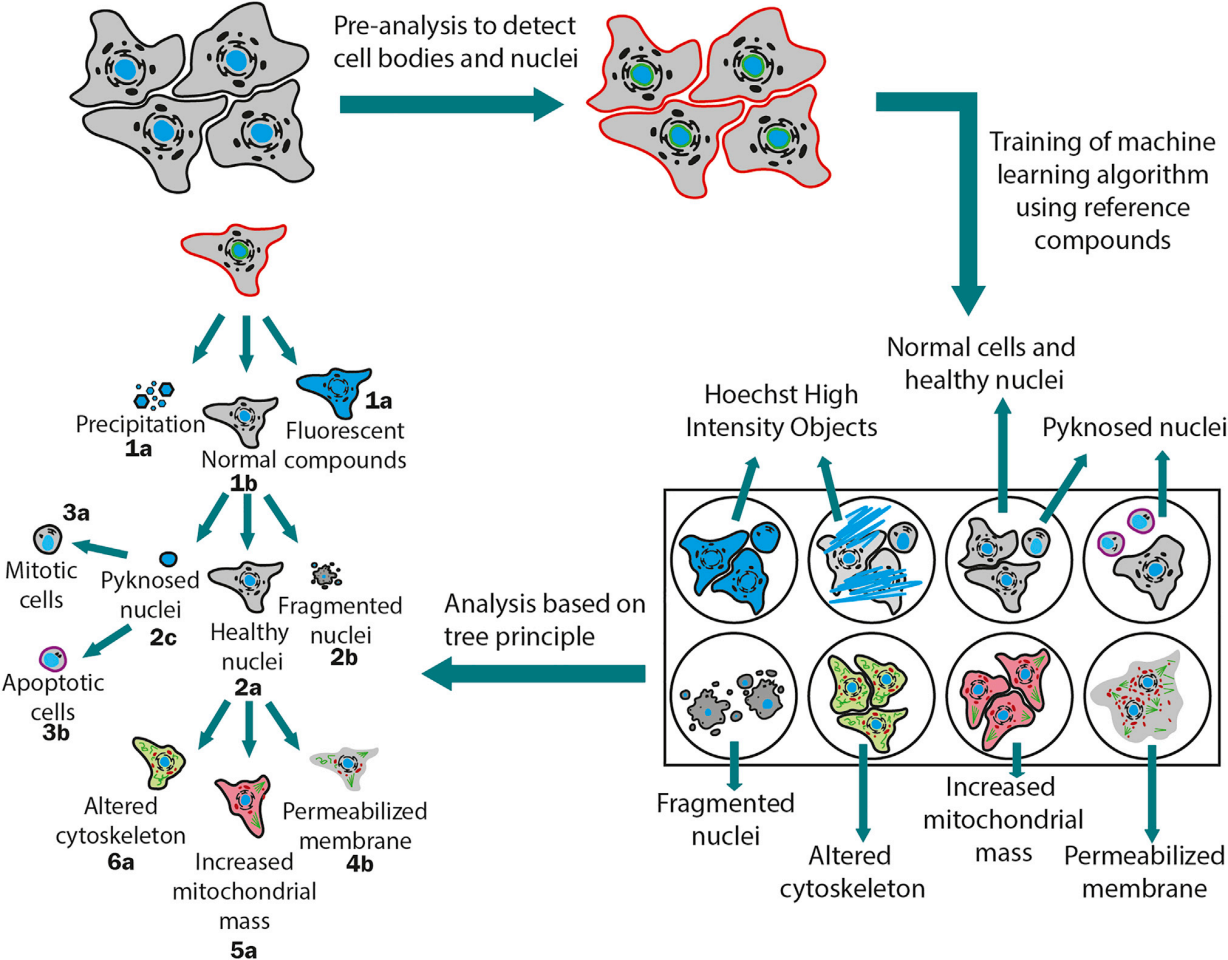
Workflow of machine learning based analysis using the CellPathfinder software The cell bodies and cell nuclei are detected in the pre-analysis of the software. Afterwards the machine learning based algorithm can be trained based on the reference compounds (see preparation of compounds and plate layout). With the trained protocol, the whole plate and all time points can be analyzed based on the tree principle explained in Tjaden et al. (Tjaden et al., 2022).
34. Use the reference compounds with known phenotypes to train the machine learning -based analysis (see Table 2).
  a. For each gating step, individual cells (minimum 10 images) are selected to serve as an example for the gating phenotype (Figure 7). This can be done by a trained scientist or the example images provided here can be used.

**Figure 7:**
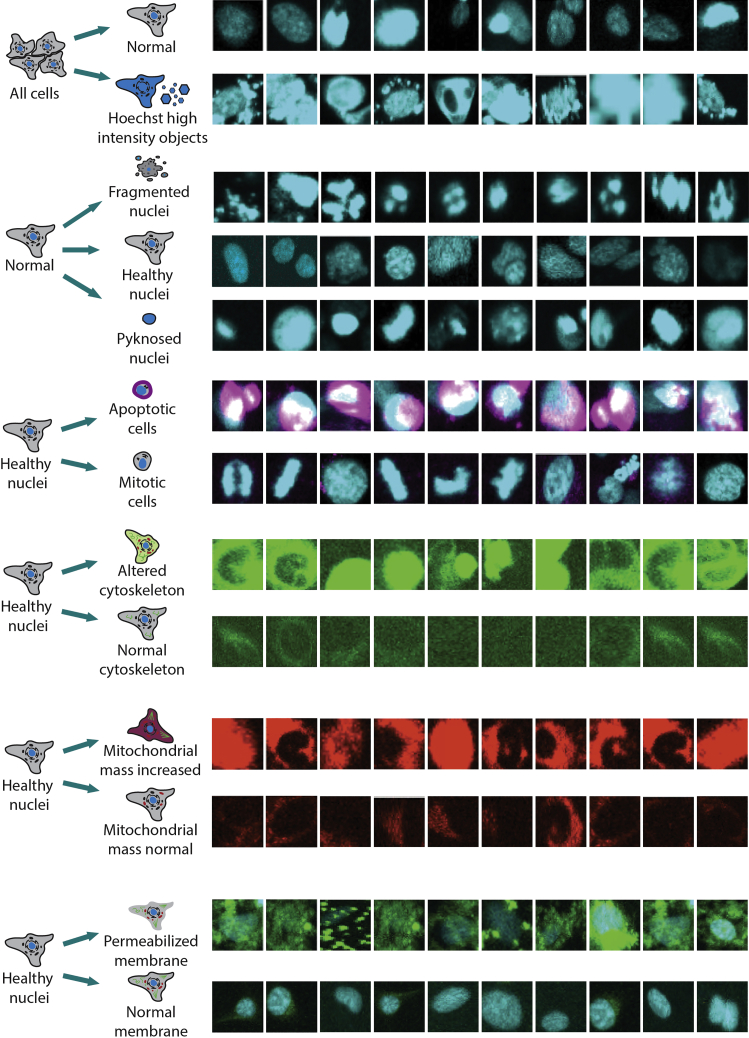
Machine Learning based algorithm training images For every gating step, 10 example images of the chosen cells are shown here. The images show the most relevant fluorescent channels for the related gating step. The cells were selected by a trained scientist.
35. After selecting the example images for the ML-based analysis, safe your analysis and run it once on all other wells containing reference compounds.
36. Ensure that the example images worked appropriately to create a robust analysis by comparing the results of all reference compound treated wells (see problem 9). The results should be consistent with the known biological mode of action of the reference compounds and should not differ from each other by more than 10%.
37. If you are sure that the machine learning-based analysis has been well trained (no deviation of the results of the reference compounds by more than 10%), the whole plate can be analyzed.
38. Your results in from of separated csv files can be found in the folder containing the data under “…\PFAnalysis” from where it can then be further evaluated (see evaluate your data).

### Optional: Generation of processed images

**Timing: 2–3 h**

**Figure 8:**
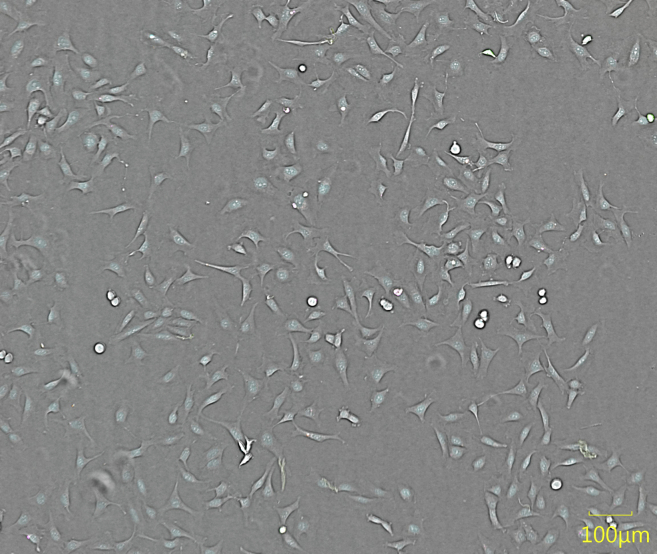
Processed Image of U-2 OS cells Brightfield confocal image of stained (blue: DNA/nuclei, green: microtubule, red: mitochondria content, magenta: Annexin V apoptosis marker) U-2 OS cells after 24 h of exposure to 0.1% DMSO.

This is an optional step. For publication and/or deposition of your data, we recommend generating processed images of your experiments (see Figure 8).

39. Open the “View” tab on the CellPathfinder software.
40. Use the contrast enhancement to adjust channel intensity levels for your image export.
41. Save Images as .jpg in a folder called “processed images”.
42. name the files according to the experimental-ID, cell line and time point e.g., CQ1-ctf006-U2OS-0h.

### Document your data

**Timing: 1 h**

This step describes how to document the generated data so it can be evaluated automatically in the next step. For preparation of your computer refer to preparation of the data analysis workflow.

43. Visit the “root path” (see preparation of the data analysis workflow) on your computer.
44. Open the metadata template file found here: Zenodo: https://doi.org/10.5281/zenodo.6325622 (excel file with multiple sheets) called “metadata_template.xlsx”. Do not change any column headings. You are free to add additional columns.
45. Fill all sheets.
  a. In the sheet “experiments”, describe your basic experimental setup.
  b. In the sheet “imaging campaigns”, describe all time points for which you acquired images. Make sure to fill the column “cq1 analysis available in folder”; this can be a local, absolute path on your computer (e.g., “C:/Users/CQ1/experiment/PFAnalaysis/timestamp_protocol/") or can be indicated relative to the “root path” (e.g., “../../experiment/PFAnalaysis/timestamp_protocol/”).
    ***Note:*** This cannot be a network path. If necessary, copy the files from a network path to your computer. See also Section “data analysis CellPathfinder software” above.
  c. The sheet “exclude from file list” is only relevant if you want to deposit your data. In that case, you can omit individual processed images from the autogenerated file list.
  d. In the sheet “compounds”, describe all compounds you used in the experiment, e.g., with their chemical structure formula as SMILES. It is recommended to include InChIs, too.
  e. In the sheet “compound batches”, list all specific batches of the compounds you used. Information may include internal identifiers and supplier lot numbers.
46. Each experiment is linked from the sheet “experiments” to a specific “compound map”. This “compound map” describes the compound batches in individual wells, as discussed in Section “compound treatment”. Example:
  a. Let us assume that you entered “multiplex1” as the value of “compound map see corresponding excel table” on sheet “experiments”.
  b. Create (or rename) a compound map sheet to “compound map multiplex1”. If you entered “multiplex2”, you should name it “compound map multiplex2”.
  c. In the sheet “compound map multiplex1” (or alike), fill out information about the concentration of the compound and of your intent, i.e., its experimental type:
    i. “blank”: a well with 0.1% DMSO.
    ii. “cells only”: a well without any addition apart from cells.
    iii. “chemogenomic candidate”: a well with a compound to be tested.
    iv. “control”: a well with a reference compound.

***Optional:*** If you want to deposit your data, fill out “raw data available in zip file” and “processed images available in folder”, but relative to the folders in BioImage Archive (see also Section “optional: deposit your data” below).

47. Save the file under a name which will be denoted “METADATA_FILENAME” below.
48. Perform automated checks on the file to make sure that it is intact: Open the Anaconda Prompt and enter:

> python quality_control_of_excel_file.py METADATA_FILENAME

49. Make sure to replace “METADATA_FILENAME” with the corresponding filename. If you saved the file under the name “overview.xlsx”, the correct command is:

> python quality_control_of_excel_file.py overview.xlsx

50. If you receive error messages, correct your file accordingly. If the error message appears to be cryptic to you, contact the authors of this protocol. If you do not see any error messages, your file is ready for the next steps.

### Optional: Deposit your data

**Timing: Multiple days, depending on your upload rate**

This is an optional step. However, we strongly recommend depositing your image data into a public repository, preferentially EBI’s BioImage Archive (https://www.ebi.ac.uk/bioimage-archive/).

51. Zip each folder of collected raw data. Each folder is typically a single timepoint of a single plate with a size of 160 GB. Zipping can take up to 1 h per folder, and results in a zip file with a size of 60 GB. Running multiple processes at the same time can prolong the zipping time.
52. Register yourself with BioImage Archive: https://www.ebi.ac.uk/bioimage-archive/
53. Start a new submission in BioImage Archive. Fill all required fields.
54. Transfer all zip files, as well as the processed images (see optional: generation of processed images) into your personal area in BioImage Archive.
55. Create file lists for the raw data and the processed images.
56. Open the Anaconda Prompt, and execute:

> python create_filelist_raw_cq1.py METADATA_FILENAME

57. Check the Prompt for error messages and, if applicable, act on them.

***Note:*** Make sure to replace “METADATA_FILENAME” with the actual filename (see Section “document your data” above).

58. You will find a new folder called “filelists_raw” in the “root path”, if all went well, and a file called “filelist_raw_data_cq1.xlsx” within that folder.
59. In the Prompt, execute:

> python create_filelist_processed_images_c1.py METADATA_FILENAME

60. Check the Prompt for error messages and, if applicable, act on them.

***Note:*** Make sure to replace “METADATA_FILENAME” with the actual filename (see “document your data” above).

61. You will find a new folder called “filelists_cq1” in the “root path”, if all went well, and a file called “filelist_cq1_processed_images_all_experiments.xlsx” within that folder.
62. Create two Study Components in your submission to BioImage Archive.
63. Use the “filelist_raw_data_cq1.xlsx” to populate a Study Component about the raw data.
64. Use the “filelist_cq1_processed_images_all_experiments.xlsx” to populate a separate study component about the processed images.
65. Submit your study in BioImage Archive.

### Evaluate your data

**Timing: 1–2 h**

This step describes the automated combination of data and metadata to derive the phenotypical characteristics and how to final evaluate each tested compound.

66. Open the Anaconda Prompt and execute:

> python merge_and_evaluate_cq1_csv_files.py_METADATA_FILENAME

67. Make sure to replace “METADATA_FILENAME” with the corresponding filename (see “document your data”).
68. Check the output for error messages and act accordingly.

69. You will find a new file called “cq1_evaluated_all.xlsx” in the “root path”. Open it to manually inspect the results. An example of the layout can be found in the Table S3 (Zenodo: https://zenodo.org/record/7092795#.YyhponZByUk). The ratios used for validation were calculated by the following equations:
  *1a = Ratio Hoechst High Intensity Objects*
  *= Cell_Count_Cell_Stats_HighIntObj/ Cell_Count_Cell_Stats*
  *1b = Ratio Normal Cells*
  *= Cell_Count_Cell_Stats_Normal / Cell_Count_Cell_Stats*
  *2a = Ratio Healthy Nuclei*
  *= Cell_Count_Cell_Stats_HealthyNuc/ Cell_Count_Cell_Stats_Normal*
  *2b = Ratio Fragmented Nuclei*
  *= Cell_Count_Cell_Stats_FragNuc/ Cell_Count_Cell_Stats_Normal*
  *2c = Ratio Pyknosed Nuclei*
  *= Cell_Count_Cell_Stats_PyknoNuc/ Cell_Count_Cell_Stats_Normal*
  *3a = Ratio Mitotic cells*
  *= Cell_Count_Cell_Stats_mitosis/Cell_Count_Cell_Stats_PyknoNuc*
  *3b = Ratio Apoptotic cells*
  *= Cell_Count_Cell_Stats_apoptosis/ Cell_Count_Cell_Stats_PyknoNuc*
  *4a = Ratio Intact membrane*
  *= Cell_Count_Cell_Stats_membrane intact/Cell_Count_Cell_Stats_HealthyNuc*
  *4b = Ratio Permeabilized membrane*
  *= Cell_Count_Cell_Stats_membrane permeab/ Cell_Count_Cell_Stats_HealthyNuc*
  *5a = Ratio Mitochondrial mass increased*
  *= Cell_Count_Cell_Stats_mito mass high/ Cell_Count_Cell_Stats_HealthyNuc*
  *5b = Mitochondrial mass normal*
  *= Cell_Count_Cell_Stats_mito mass normal/Cell_Count_Cell_Stats_HealthyNuc*
  *6a= Tubulin effect*
  *= Cell_Count_Cell_Stats_tubulin effect/Cell_Count_Cell_Stats_HealthyNuc*
  *6b = Tubulin normal*
  *= Cell_Count_Cell_Stats_tubulin normal/ Cell_Count_Cell_Stats_HealthyNuc*
  a. Each row corresponds to the data of one compound at a certain timepoint
  b. All rows contain all information from the sheets in the metadata file, i.e., from the compound map, the compound batch, the compound, the imaging, and the experiment. This information can be used to filter down to entries of interest by standard spreadsheet software.
  c. The cell counts in each row are automatically compared to the mean of all wells in the same plate at the same timepoint marked as “blank” according to their “experimental type” (see “document your data”). This allows you to judge whether the phenotype is unique to the compound or generic to the experimental setup.
  d. As an example, we have implemented the ratio of the cell count per well for cells annotated as “healthy” and the mean of cell counts for cells annotated as “healthy” across all wells containing 0.1% DMSO (i.e., the “blank”). You can use this metric as a rough proxy of cytotoxicity.
70. Compare the values of interest for individual “blank” rows against the mean of all “blanks”. This gives you a feeling for “natural distribution” of values for an “uninteresting” effect.

***Note:*** Taking the sample data provided with this protocol, the range of values for the ratio of healthy cells (described in the step above) is approx. 0.6–1.2 for individual “blank” rows, with an average of approx. 0.9 and a standard deviation of 0.3. You can use this information to define thresholds to alarm you on “interesting” compounds.

71. We also implemented a measure on “relative growth”, comparing information from individual wells with compound-treated cells against the mean of “blank” wells.
  ***Note:*** More measures can easily be implemented in the Python script by interested researchers. You are also invited to contact the authors of this protocol.
  a. The “relative growth” was calculated by the following equation (Hafner et al., 2016):

$$relative\;growth=2^{\frac{\text{log}2\left(\frac{atx}{at0}\right)}{\text{log}2\left(\frac{btx}{bt0}\right)}}-1$$

a = normal cell count compound.

b = normal cell count DMSO 0.1%.

tx = timepoint of interest.

t0 = timepoint after 0 h.

We defined threshold values (see Table 4), that will mark a compound with a comment. If a compound is marked in more than one cell line it should be flagged and the property should be evaluated in the context of the compound (target, concentration, IC50 etc.).

**Table 4:** Thresholds of compound properties

Property group	Property name	Threshold
Compound properties	Hoechst High Intensity Objects **1a**	> 50%
Cell viability properties	Healthy Nuclei **2a**	< 50%
	Fragmented Nuclei **2b**	> 50%
	Pyknosed Nuclei **2c**	> 50%
Phenotypical properties	Tubulin effect **6a**	> 50%
	Mitochondrial mass **5a**	> 50%
	Membrane permeabilized **4b**	> 50%

## Expected outcomes

**Figure 9:**
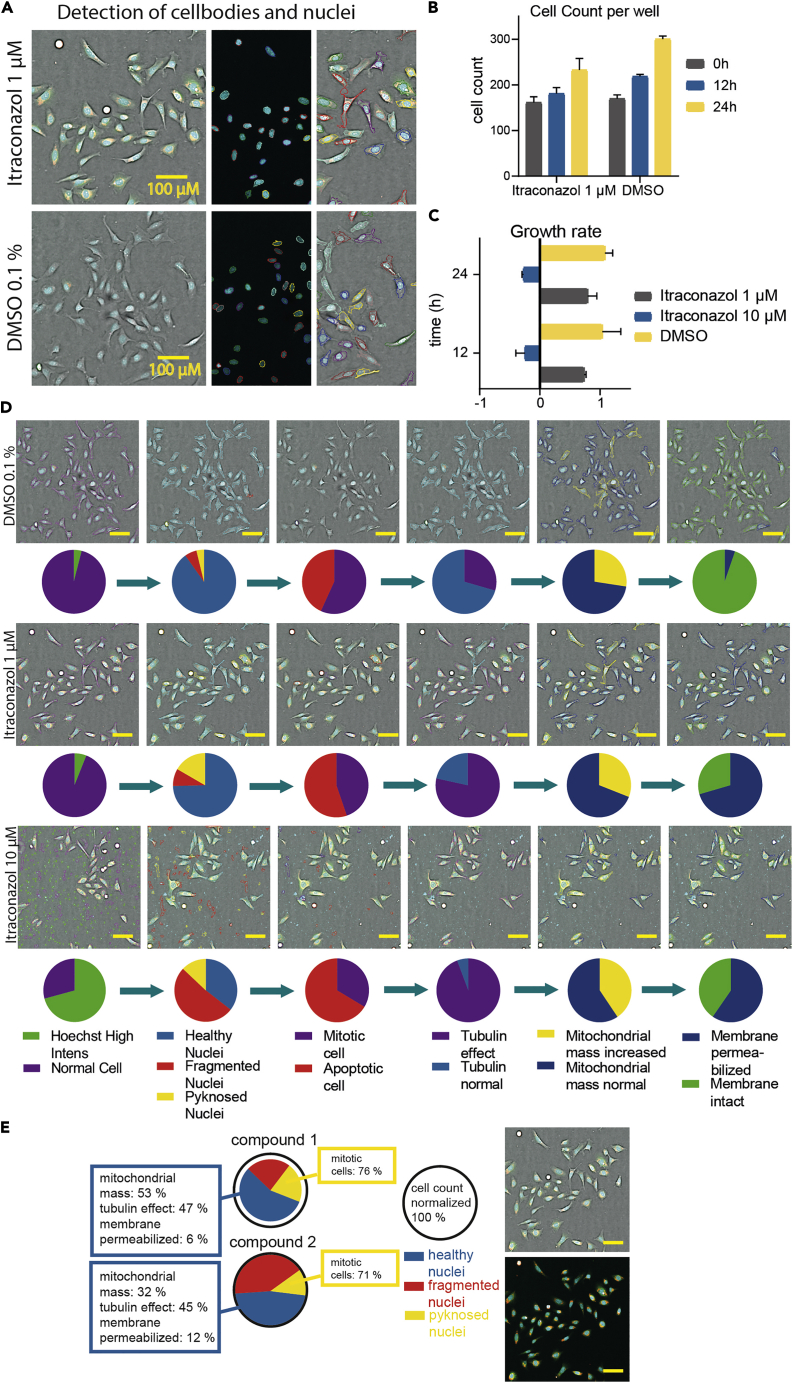
Expected outcome after multiplex assay based on the example of Itraconazol (A) Processed Image of stained (blue: DNA/nuclei, green: microtubule, red: mitochondria content, magenta: Annexin V apoptosis marker) U-2 OS cells after 24 h of exposure to Itraconazol (1 μM) and 0.1% DMSO and detection of nuclei and cell bodies after analysis using the CellPathfinder software. (B) Detected cell count per well after 0 h, 12 h and 24 h of compound exposure (Itraconazol 1 μM) in comparison to cells treated with 0.1% DMSO. Error bars show SEM of two biological duplicates. (C) Calculated relative growth called “growth rate” after 12 h and 24 h of compound exposure (Itraconazol 1 μM and DMSO 0.1%) to U-2 OS cells.Error bars show SEM of two biological duplicates. (D) Gating of U-2 OS cells using the CellPathfinder analysis after exposure to 10 μM and 1 μM of Itraconazol in comparison to 0.1% DMSO after 24 h. Pie charts show the ratios of the different gating steps. Scale bars show 50 µm. (E) Example figure for compound annotation after multiplex assay including gating ratios as well as two processed images of stained U-2 OS cells (blue: DNA/nuclei, green: microtubule, red: mitochondria content, magenta: Annexin V apoptosis marker).

The aim of this protocol is the improved annotation of the cell health effect of a compound in a high-content manner. It is based on nuclear morphology and additional phenotypic characteristics such as tubulin effect, mitochondrial mass or membrane permeabilization. A detailed outcome exemplified by Itraconazol tested at 10 μM and 1 μM in comparison to DMSO 0.1% is shown in Figure 9.

After detection of cell bodies and nuclei using the CellPathfinder software (see data analysis CellPathfinder software) processed images (see optional: generation of processed images) or fluorescent images can be generated (Figure 9A). In order to assess the viability of the cells at a certain time point, the cell count per well (Figure 9B) or calculated growth rate (Figure 9C) can be shown. The growth rate serves as an assessment of whether a compound has cytostatic (GR 1-0) or cytotoxic effects (GR < 0) (Hafner et al., 2016). Further compound annotation is achieved by various additional gating steps (see data analysis CellPathfinder software). The ratios of compounds and controls can be represented in different ways e.g., pie charts (Figure 9D).

For automated compound annotation, certain thresholds regarding the gated properties were defined as shown in Table 4). It is nevertheless advisable to manually double check images and raw data before excluding a compound based on these thresholds. An example for compound annotation can be found in Figure 9E).

Further examples of outcomes from this protocol are demonstrated in Tjaden et al.(Tjaden et al., 2022), where 215 compounds were tested successfully using the protocol described here.

## Limitations

The described Multiplex assay contains some complex steps, which is why accurate performance of described working steps and data processing are necessary for data acquisition and analysis. Here, we describe the protocol for adherent U-2 OS cells. The protocol was also tested for the adherent cell lines HEK293T and MRC-9 fibroblasts (Tjaden et al.) (Tjaden et al., 2022). For other cell lines, adjustment of the measurement protocol as well as the analysis protocol for the CellPathfinder software might be necessary. The application of suspension cells or 3D culture was not tested and must therefore be carefully reviewed before using these cell lines.

Image acquisition of 384 well plates with multiple time points requires rather extended data storage capacity and computational processing power. We advise against saving the imaging data directly to a network connection, resulting in longer and not stable measuring time periods, which could bias the final results. Saving the data first locally on internal/external storage space followed by a data transfer to the suitable storage server is therefore suggested.

The measurement time for one plate determines the frequency of data acquisition possible. Therefore, time points cannot be chosen at too short intervals if a whole 384 well plate is measured. There are also temporal deviations between the first and the last measured well. We recommend therefore including the reference compounds at different plate areas to correct for this effect. For very high frequency data acquisition (<1–2 h) one can limit the number of imaged wells or adapt the image acquisition protocol.

In the workflow described here, all compounds are tested at 10 μM and 1 μM. Depending on target specificity and mechanism of action of the compounds, these concentrations can be adjusted.

The protocol is performed using a 10× magnification. For more detailed phenotypical readouts such as structure differentiation of tubulin or localization of mitochondria, higher magnification should be used. The magnification used in the protocol described here enables therefore only limited detection of intracellular spatial information.

The detection of cellular features in this protocol relies extensively on fluorescence intensity measurements. This is essential for the detection of nuclei using Hoechst33342 which enables the further gating steps based the shape of cellular nuclei. Compounds that interfere with light absorption such as fluorescent compounds, may interfere with this protocol and result in a high ratio of “Hoechst High Intensity Objects”.

Compounds, insoluble in DMSO and thus precipitated in their DMSO stocks can result in false negative outcomes (see problem 4). Chemical quality control of the DMSO stocks of tested compound using LC-MS can help detect and account for this occurrence.

## Troubleshooting

### Problem 1

A different cell line than U-2 OS is used and not properly detected and/or gated.

If different cell lines are used, the analysis protocol for U-2 OS cells is no longer applicable due to different cell morphology.

### Potential solution

The measurement protocol as well as the analysis protocol should be adjusted accordingly. We tested two other adherent cell lines (HEK293T and MRC-9 fibroblasts), where we could see that mostly the cell body detection and the machine-learning based algorithm must be changed due to the different overall cellular morphology of the different cell lines. For the adherent cell lines HEK293T and MRC-9 fibroblasts, the adjusted protocol can be found here: Zenodo: https://doi.org/10.5281/zenodo.6415330. Other cell lines, especially suspension cells, have not been optimized or tested (see limitations).

### Problem 2

Cell seeding density is not optimal. Either too little cells are observed in each image or high confluency impedes the segmentation of individual cells.

### Potential solution

Optimize the cell seeding density as described in steps 2–5 in section optimize cell seeding density. Proper assessment of the cell viability and cell passaging at a confluence not higher than 90% can increase robustness and reproducibility of this assay. As mentioned above an optimal confluence 24 h after seeding should be 90%. If the cell density cannot be optimized by the cell seeding number, the imaging time points could be prolonged or shortened to obtain better results.

### Problem 3

Evaporation of the medium.

Certain wells, especially on the plate edges may contain less liquid and a higher apoptosis rate can be observed in control wells located at the edge of the 384 well plate.

### Potential solution

Increased evaporation of cell medium can affect the outcome of the experiment by changing the composition of medium components and increasing the concentration of tested compounds.

As shown in the layout (see Figure 2), it is best practice to exclude the outer wells of the 384 well plate from the measurement and fill those with 100 μL PBS buffer or water (see addition of fluorescent dyes and cell seeding).

### Problem 4

Compounds are not soluble in DMSO and/or precipitation of compounds on LDV source plate.

Compounds that are insoluble in DMSO or less soluble than others can either not be dispensed by the used ECHO Labcyte protocol to the cells or can precipitate in the source plate.

### Potential solution

DMSO solubility of compounds should be tested beforehand. If not soluble in DMSO, try diluting in water and pipet by hand or with ECHO Labcyte protocol for aqueous solvents (384LDV_aq). Look by eye to see if the compound precipitated on the Echo plate before pipetting. LC-MS can help detect and account for this occurrence. Frequent freeze-thaw-cycles can increase this problem.

### Problem 5

Cells are not evenly distributed within the well.

An uneven distribution of cells can lead to suboptimal cell growth under the assay conditions or a falsely overestimation of the compound toxicity. Additionally, a lower number of cells compromises the statistical analysis and can impede the clarity of the results.

### Potential solution

Before testing the cells, make sure that the cells have the optimal cell seeding density and are detached by trypsin treatment to avoid clusters (see optimize cell seeding density). Gently mix the cells before seeding to ensure an even distribution of the cells in the culture medium. The waiting step described in 6 is important to avoid any strong convection currents within the wells due to the different temperature of the medium and the surface of the plate. The waiting period allows cells to start attaching themselves to the plate surface and makes them less susceptible to this phenomenon when placed subsequently in the incubator.

If the majority of the cells are found on the outer rim of the wells of an already prepared plate, one can change the image acquisition spot of the well at the CQ1 protocol to a spot less centered.

### Problem 6

The focus of CQ1 protocol is set incorrectly.

If the focal plane of the microscope has not been properly adjusted, cells may not be detected and unwanted errors due to focusing on dirt on the plate may limit the measurement. This can happen when a different plate, not implemented in the CQ1 software is used, or if the plate is uneven or if there is evaporation on the plate.

### Potential solution

The plate dimensions of the used plates should be implemented in the CQ1 software. Make sure you can adopt the temperature of the microscope to avoid condensation occluding the imaging and to prevent focus problems due to evaporation on the plate lid. To set the right focus, test the autofocus of the CQ1 on your plate using Channel 1. If the focus area differs from the already set focus, change the protocol accordingly.

### Problem 7

The results of the reference compounds after the pre-analyzing using the machine learning-based algorithm differed by more than 10%.

After the pre-analysis the results from the reference compounds should not differ by more than 10%. This can happen when the normal growth conditions are affected e.g., due to evaporation (see problem 3), not evenly distribution (see problem 5), different cell morphology due to overgrowing of the cells or the fluorescence intensity levels differed from the training set. If so, the machine-learning-based algorithm should be adjusted accordingly.

### Potential solution

The machine learning-based algorithm has to be trained for every new experiment. The same algorithm can then be used for the following experiments with the same conditions. To check if the analysis worked appropriately, the reference compounds are added in quatruplicates to double-check your results (see steps 36–39 in data analysis CellPathfinder software). If the results of the reference compounds differ from each other more than 10%, the machine learning algorithm should be trained again with new example images. For this, follow steps 34–36 of Data analysis CellPathfinder Software. Example training images can be found in Figure 7.

## Resource availability

### Lead contact

Further information and requests for resources and reagents should be directed to and will be fulfilled by the lead contact, Susanne Müller-Knapp (susanne.mueller-knapp@bmls.de).

### Materials availability

This study did not generate new unique reagents.

## Data Availability

The images and raw datasets generated during this study are available at BioImage Archive: https://www.ebi.ac.uk/biostudies/studies/S-BIAD145. Analysis data and codes reported in this paper can be found at Zenodo: https://doi.org/10.5281/zenodo.6325622. All supplemental data have been deposited to Zenodo database under the following link: Zenodo: https://zenodo.org/record/7092795#.YyhponZByUk. Original data for figures in the paper are available at Tjaden et al. (Tjaden et al., 2022).
